# The association of socio-demographic and environmental factors with stunting among under-five children in Hawassa City, Sidama National Regional State, Ethiopia

**DOI:** 10.1017/jns.2022.29

**Published:** 2022-05-05

**Authors:** Berhanu Kibemo, Afework Mulugeta, Dejene Hailu, Baye Gelaw

**Affiliations:** 1 School of Nutrition, Food Science and Technology, College of Agriculture, University of Hawassa, Hawassa, Ethiopia; 2 Department of Nutrition and Dietetics, School of Public Health, College of Health Sciences, Mekelle University, Mekelle, Ethiopia; 3 School of Public Health, College of Medicine and Health Sciences, Hawassa University, Hawassa, Ethiopia; 4 Department of Medical Microbiology, School of Biomedical and Laboratory Sciences, College of Medicine and Health Sciences, University of Gondar, Gondar, Ethiopia

**Keywords:** Chronic undernutrition, Environmental factors, Hawassa city, Preschooler children, Socio-demography

## Abstract

Undernutrition in children is a challenging problem in developing countries, including Ethiopia. Stunting is the most prevalent form of undernutrition. The majority of studies on childhood stunting and its associated factors focused on children, maternal and socioeconomic components. However, a few studies reported poor WaSH status and antibiotic exposure as environmental risk factors for child stunting, and the case of socio-demographic factors also lacks consistency. Concerning this, there is a lack of information in Ethiopia. Therefore, the present study assessed the association of socio-demographic, WaSH, and antibiotic exposure with stunting among under-five children. A cross-sectional study was conducted involving 340 mother–child pairs. Anthropometric data were collected using standard and calibrated height and weight scales. For factorial data, an interviewer-guided standard questionnaire was used. Logistic regression analyses were used to identify factors determining childhood stunting. In the present study, the prevalence of stunting, underweight and wasting was 14⋅7 % (95 % CI 10⋅9, 18⋅5), 4⋅4 % (95 % CI 2⋅4, 6⋅8) and 2⋅1 % (95 % CI 0⋅6, 3⋅5), respectively. Low dietary diversity, being born from a mother with an education level of secondary school, and belonging to a female-headed household were positively associated (*P* < 0⋅05) with stunting. The prevalence of overall undernutrition was lower (21⋅2 %) in the study area. Stunting was significantly associated with dietary diversity, maternal educational level and sex of households head. The government policy should focus on enhancing the dietary diversity of households, and encouraging women's education.

## Introduction

Childhood undernutrition is widespread in low- and middle-income countries. In these countries, it is an important and indirect cause of child mortality. Undernutrition in children is among the challenges in developing countries like Ethiopia^(1)^. Stunting is a result of a longer period of deprivation of essential body nutrients, while wasting occurs when there is a shortage of nutrients, either due to diarrheal episodes or dietary deficiency for a short period of time. Underweight might prevail as a consequence of both long-term and short-term exposure to adverse nutritional conditions^(2)^.

Over the last few decades, there have been global advances in reducing stunting in children, but those advances have been inconsistent. Globally, there were 252⋅8 million stunted under-five children in 1990, whereas the number of stunted children was reduced to 144 million in 2019^(3)^. Globally, although there has been a reduction in childhood stunting over the last decades, it has been a challenging problem for Africa; particularly the eastern part of the continent. Ethiopia is included in the list of African countries where the most prominent prevalence of stunting was reported^(4,5)^. Indeed, the country has achieved a decrease in undernutrition in the past 14 years due to the corporate effort of governmental and non-governmental bodies in fighting against malnutrition^(2,4)^. A decrease in undernutrition was reported in Tigray from 41 to 39⋅3 %, in Amhara from 56⋅6 to 46⋅3 %, in Oromia from 41 to 36⋅5 %, and in the South Nation Nationality and People Region (SNNPR) from 51 to 38⋅6 % during the years 2005–2016. Stunting rates decreased from 51 to 38 %^(5,6)^. Despite these achievements, however, the prevalence of stunting among under-five children in Ethiopia as a nation demands a huge investment^(7)^.

The lack of nutritious food in the early stages of life can result in metabolic disorders and mortality, as well as a weakened mental and psychological state. The impact of youngster's unhealthiness is enduring and goes well past adolescence. For example, a lack of nutritious food at a young age reduces educational performance and work capacity and increases the risk of chronic illnesses later in life^(8,9)^. In Ethiopia, two out of three children's deaths are related to malnutrition^(10)^. In the past two decades in Africa, different studies have been done on the prevalence of childhood undernutrition and its associated factors. According to the reports, the prevalence of undernutrition was high and linked to child, maternal, dietary and socioeconomic factors^(11,12)^. The same findings have been documented in Ethiopia, focusing on rural communities^(13,14)^. On the other hand, recently, researchers have been inspired by a new scientific view as the predictor of childhood undernutrition, which targets the gut microbiota. The view is dealing with socio-demographic and environmental factors that involve a mode of delivery, antibiotic exposure, duration of breast-feeding, dietary fibre consumption and WaSH status^(15)^. The formative starting points of wellbeing and illness theory suggest that a socio-demographic and environmental factors window exists where ecological openings, including the method of birth, balanced diet, breast-feeding, disease and antibiotics, lead to programming impacts that can influence long-term wellbeing^(16,17)^.

A few studies reported that poor WaSH status and antibiotic exposure as environmental risk factors for childhood stunting, and the case of socio-demographic factors also lacks consistency^(18,19)^. Some studies reported a significant association between WaSH and stunting, and the others found no association^(20–23)^. Presently, antibiotic exposure is also getting attention in association with children under-five nutritional status^(24–27)^. Concerning this, however, there is a lack of information in Ethiopia. Therefore, the present study investigated the association of socio-demographic and environmental factors with stunting among children under 5 years of age at Hawassa City, Sidama National Regional State, Ethiopia.

## Materials and method

### Study area and period

This study was conducted at Hawassa, the capital city of the Southern Nation, Nationalities, and Peoples Region (SNNPR), and Sidama National Regional State. Hawassa is located at 7°3′0″N latitude and 38°28′0″E longitude. The city is 270 km away from Addis Ababa, the capital city of Ethiopia. The metropolis is divided into 8 subcities and 32 kebeles. According to the document of the housing and population census, the projected population of Hawassa metropolis administration in 2011 was 329 734, out of which 169 677 were males and 160 057 were females^(18)^. The study was conducted from April 2021 to August 2021.

### Source population and study design

The source population for the present study was all children aged 6–59 months and living at Hawassa City, Sidama Regional State. A cross-sectional study design was employed to collect the data.

### Sample size determination

The sample size was determined using G-power by considering the multivariable logistic regression test, an effect size of 0⋅07, *α* 0⋅05, *β* 0⋅10 and 13 independent variables. The sample size calculated was 332, but by using a 5 % non-response rate, it was finally determined 349. However, in the present study, a total of 340 mother–child pairs were included, which implies a 97⋅42 % success rate.

### Sampling technique

The primary sampling unit was the subcity of Hawassa, while the households in the subcity were the secondary sampling units. The tertiary sampling units, the mother–child pairs existing in the households, were selected through a simple random sampling method. According to the Hawassa Health Office 2021 report (unpublished), the four subcities (Tabor, Menaharia, Tula and Hayek Dar) had higher numbers (450–867) of under-five children. During data collection, participants were recruited and enrolled on a voluntary basis.

### Inclusion and exclusion criteria for selecting mother–child pairs

Children with the following inclusion criteria were eligible for the present study: age of 6–59 months, the beginning of solid food intake, and food intake in addition to breast-feeding. Children with any of the exclusion criteria were excluded from the study: any gastrointestinal or underlying pathology, any chronic illness and diarrheal disease related to travel history within a month before the study.

### Data collection tools and procedures

For anthropometric data collection from children, permission was obtained from the households. Data were collected from children aged 24–59 months by measuring their weight in underwear and without shoes with an electronic scale (Type SECA 861 or SECA 813, Hamburg, Germany) to the nearest 0⋅1 kg and their height in the Frankfort plane with a telescopic height instrument (Type SECA 225 or SECA 214) to the nearest 0⋅1 cm. Data from children aged 6–23 months, on the other hand, were collected by measuring the child's weight and that of the mother/caregiver, then subtracting the weight of the mother/caregiver from the sum weight of the child and the mother/caregiver. The height was measured by using an accurately graduated length board and was recorded to the nearest millimetre. The age of the children was obtained from a parental recall using an events calendar. The height and weight of the children were measured twice, and the average was taken. In each case, the measuring instruments were calibrated at least twice a day. According to the World Health Organization (WHO), wasting, stunting and underweight are defined as *Z*-scores of less than −2 standard deviations of weight for height, height for age and weight for age, respectively^(28)^. An interviewer-administered questionnaire was used after a 5 % sample pre-test in the Philedefia kebele, Addis ketema subcity which was not a part of the study areas. The questionnaire was translated into the Amharic language, the official language of Ethiopia, and the data collectors were fluent speakers of Amharic. The respondents were at least capable of speaking and listening to Amharic. The collected data was translated back to English by a proficient translator to ensure accuracy and consistency. The data collection tool for dietary diversity score was adapted from the World Health Organization's guidelines proposed for assessing infant and young child feeding practices. Eating of four or more of the seven food groups means that the child is probably to consume at least one animal food source and at least one fruit or vegetable in addition to the staple food (grains, roots or tubers) in the last 24 h. Four food groups should be drawn from the list of seven food groups: grains, roots and tubers, legumes and nuts; dairy products (milk yoghurt, cheese); meat, fish, poultry, and liver/organic meat; eggs; vitamin A-rich fruits and vegetables; and other fruits and vegetables^(12)^. Data were gathered from the mother's or caregiver's recall of foods given to the child in the past 24 h before the interview. Moreover, data on household sanitation and hygiene, a child's mode of delivery, breast-feeding practice and taking antibiotics were collected. Breast-feeding practice was assessed based on the beginning of complementary feeding at 6 months, continued breast-feeding up to 2 years, colostrum feeding and exclusively breast-fed for 6 months. It was rated as ‘optimum’ if all those conditions were fulfilled, and ‘suboptimal’ if one or more of those were missing. Sanitation and hygiene status was assessed according to the WaSH guidelines for field practitioners^(29)^. For antibiotic exposure, the mother/caregiver was asked about the taking of any antibiotics by the child for the past 3 months before the survey.

### Data analysis

Before the data processing, each questionnaire was checked for completeness and consistency. Then, the data were coded, entered and cleaned using SPSS version 20⋅0 (SPSS, Inc., Chicago, IL, USA). Eventually, the data were analysed by using bivariate and multivariate logistic regression to identify predictors of stunting among children.

### Ethical consideration

This study was conducted according to the guidelines laid out in the Declaration of Helsinki, and all procedures involving human subjects were approved by the Institutional Review Board of Hawassa University (Ref. No.: IRB/086/13). Verbal informed consent was obtained from all subjects before data collection. Verbal consent was witnessed and formally recorded.

## Results

### Socio-demographic and environmental characteristics of child–mother pairs

The proportion of male and female children enrolled in the present study was almost equal; 50⋅3 % and 49⋅7 %, respectively. The median (IQR) age of the children was 29⋅5 (24) months. Of the total participants, 88 (25⋅9 %) children were born through caesarean section (CS). Data on breast-feeding practice showed that 115 (33⋅8 %) children were exposed to suboptimal breast-feeding practice (Table 1). The present study showed that 108 (31⋅8 %) children took antibiotics to treat diarrheal disease for 1–2 courses within the last 3 months before the survey. Most of the households, 301 (88⋅5 %), had access to improved sources of drinking water supplied in their yard. Despite having water access, only 37 (10⋅9 %) households had handwashing facilities around the toilet.

**Table 1.** Socio-demographic characteristics and environment-related factors of child–mother pairs at Hawassa, Sidama Region, Ethiopia (*N* 340)

Variables	Category	Median (IQR)	*N* (%)
Age in month	6–12	29⋅50 (24)	33 (9⋅7)
	13–35		177 (52⋅1)
	36–59		130 (38⋅2)
Sex	Male	1⋅00 (1)	171 (50⋅3)
	Female		169 (49⋅7)
Mode of delivery	Spontaneous	0⋅00 (1)	252 (74⋅1)
	Caesarean section		88 (25⋅9)
Breast-feeding practice	Optimum	0⋅00 (1)	225 (66⋅2)
	Suboptimal		115 (33⋅8)
Antibiotic exposure	Exposed	0⋅00 (1)	108 (31⋅8)
	Not exposed		232 (68⋅2)
Improved source of drinking water	Yes	0⋅00 (1)	301 (88⋅5)
	No		39 (11⋅5)
Handwashing facility around the toilet	Yes	1⋅00 (0)	37 (10⋅9)
	No		303 (89⋅1)
HH's faeces and wastes management	Improved	0⋅00 (1)	176 (51⋅8)
	Not improved		164 (48⋅2)
Marital status	United	0⋅00 (1)	318 (93⋅5)
	Not united		22 (6⋅5)
Maternal education level	College and above	2⋅00 (1)	58 (17⋅1)
	9–12 grade		72 (21⋅2)
	8 grade and below		210 (61⋅8)
Family size	3–4	1⋅00 (1)	160 (47⋅1)
	5–6		139 (40⋅9)
	7–10		41 (12⋅1)
Sex of head of household	Male	0⋅00 (1)	247 (72⋅6)
	Female		41 (12⋅1)
	Sex-jointed		52 (15⋅3)

On the other hand, 176 (51⋅8 %) households disposed of their solid waste using a private facility. Data on the marital status of the families of the children showed that 93⋅5 % of the households were in the union of marriage. The educational status of the mothers of the children enrolled in the present study demonstrated that 61⋅8 % of the mothers had an educational level of 8th grade or below. A low proportion (12⋅1 %) of the households were female-headed, and the rest were male-headed and both sex-jointed-headed. Nearly half of the households had a family size of three to four members (Table 1).

### Prevalence of undernutrition among children under the age of five

**Fig. 1.**
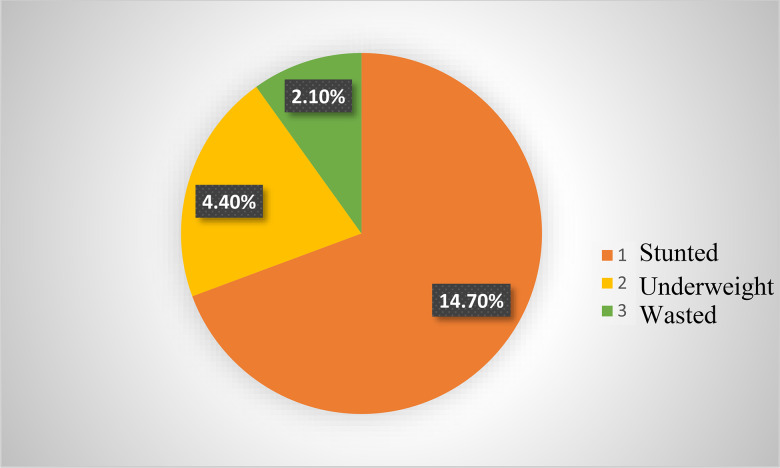
Prevalence of undernutrition among children under the age of five at Hawassa, Sidama Region, Ethiopia, 2021.

Data on the nutritional status of the under-five children at Hawassa city showed that 95⋅6 and 97⋅9 % of them were not underweight and not wasted, respectively. The overall prevalence of undernutrition among under-five children was 21⋅2 %. The prevalence of stunting, underweight and wasting was 14⋅7, 4⋅4 and 2⋅1 %, respectively (Fig. 1).

### Dietary diversity among children under the age of five

About half of the sample children consumed low dietary diversity food (48⋅5 %), followed by 1/3 consuming food with minimum dietary diversity (28⋅2 %). Relatively, a small proportion (21⋅8 %) of the children consumed moderate dietary diversity food. The number (1⋅5 %) of children who consumed high dietary diversity food was very low in the study area (Table 2).

**Table 2.** Dietary diversity among under-five children at Hawassa City, Sidama Region, Ethiopia (*N* 340)

Dietary status of the children	Frequency	Percent	Valid Percent	Cumulative Percent
Low dietary diversity score	165	48⋅5	48⋅5	48⋅5
Minimum dietary diversity score	96	28⋅2	28⋅2	76⋅8
Moderate dietary diversity score	74	21⋅8	21⋅8	98⋅5
High dietary diversity score	5	1⋅5	1⋅5	100⋅0
Total	340	100⋅0	100⋅0	100⋅0

### The association of stunting with risk factors among under-five children

Our findings did not show a significant association between the following independent variables: access to improved drinking water sources [AOR 0⋅79, 95 % CI 0⋅32, 1⋅89, *P* = 0⋅63], access to improved hygiene facilities [AOR 3⋅1, 95 % CI 0⋅69, 13⋅96, *P* = 0⋅14], sanitation [AOR 1⋅38, 95 % CI 0⋅74, 2⋅55, *P* = 0⋅3] and antibiotic exposure [AOR 1⋅65, 95 % CI 0⋅86, 3⋅18, *P* = 0⋅13], and stunting in children. While children with low dietary diversity were more likely to be stunted than those with minimum dietary diversity [AOR 2⋅98; 95 % CI 1⋅18, 7⋅51]. Maternal education up to college level or above was negatively associated with stunting [AOR 0⋅22, 95 % CI 0⋅05, 0⋅99]. Moreover, belonging to a male-headed household was negatively associated with child stunting [AOR 0⋅16; 95 % CI 0⋅03, 0⋅73] (Table 3).

**Table 3.** The association of stunting with risk factors among children aged 6–59 months at Hawassa City, Sidama Region, Ethiopia (*N* 340)

Variables	Predictors category	COR (95 % CI)	*P*	AOR (95 % CI)	*P*
Age in months	6–12	1		1	
	13–35	0⋅46 (0⋅13, 1⋅66)	0⋅24	0⋅45 (0⋅12, 1⋅66)	0⋅23
	36–59	0⋅73 (0⋅39, 1⋅36)	0⋅32	0⋅76 (0⋅4, 1⋅44)	0⋅39
		*r*^2^ 0⋅010		*r*^2^ 0⋅056	
Sex	Male	0⋅92(0⋅5, 1⋅68)	0⋅79	0⋅89 (0⋅48, 1⋅65)	0⋅72
	Female	1		1	
Antibiotic exposure	Exposed	1⋅53 (0⋅82, 2⋅84)	0⋅18	1⋅65 (0⋅86, 3⋅18)	0⋅13
	Not exposed	1		1	
Dietary diversity score (DDS)	<4	3⋅042 (1⋅21, 7⋅56)	0⋅017	2⋅98 (1⋅18, 7⋅51)	0⋅020*
	≥4	1		1	
Maternal education	9–12 grade	0⋅16(0⋅04, 0⋅69)	0⋅014	0⋅22 (0⋅05, 0⋅99)	0⋅049*
	College and above	1		1	
Sex of head of household	Female	0⋅17 (0⋅4, 0⋅65)	0⋅010	0⋅16 (0⋅03, 0⋅73)	0⋅018*
	Male	1		1	
Marital status	Not united	1⋅78 (0⋅63, 5⋅08)	0⋅28	0⋅59 (0⋅15, 2⋅33)	0⋅46
	United	1		1	
Handwashing facility near the toilet	Not available	3⋅31 (0⋅77, 14⋅21)	0⋅11	3⋅1 (0⋅69, 13⋅96)	0⋅14
	Available	1		1	
Access to drinking water	Not improved	0⋅76 (0⋅32, 1⋅83)	0⋅54	0⋅79 (0⋅32, 1⋅89)	0⋅63
	Improved	1		1	
Sanitation status	Not improved	1⋅34 (0⋅73, 2⋅46)	0⋅34	1⋅38 (0⋅74, 2⋅55)	0⋅3
	Improved	1		1	

**Key:** *P significant at *P* < 0⋅05.

## Discussion

Undernutrition is the leading cause of morbidity and mortality among under-five children. Study reports demonstrated that among the 178 million under-five children who are stunted, more than 90 % live in Africa and Asia. In Africa, the prevalence of stunting among under-five children was reported at 36 %, and the higher number of stunted children was reported in East Africa. Data of the present study showed that the overall prevalence of undernutrition was 21⋅2 % which is lower than previous reports in Hawassa City (29⋅7 %)^(19–31)^, the surrounding districts (45⋅8 %)^(32)^ and in Ethiopia (49 %)^(13)^. However, a relatively similar prevalence of undernutrition was reported in Addis Ababa (22 %)^(23,33)^.

The present study showed no statistically significant association between stunting and socio-demographic characteristics such as age and sex of the under-five children. Previous to this study, similar reports were documented that there was no statistically significant association between sex and stunting in the rural kebeles of Hawassa City^(31)^. On the other hand, different reports in Ethiopia documented that male children were more affected by chronic malnutrition than their female counterparts^(2,18,34)^. There are also other studies from different parts of Ethiopia that reported a significant association between the age of the child and stunting^(8,12,19,34–36)^. Moreover, those studies also showed the chances of a child being stunted increase as they get older. This has been mainly explained by poor child feeding practice^(37,38)^ and more exposure to environmental enteropathy accompanied by diarrhoea^(22,39)^. In the present study, the association might be influenced by the high performance (94⋅2 %) of vaccination^(31)^ in the city that enables the children to cope with some enteric pathogens^(40,41)^.

Based on the present findings, the majority (88⋅5 %) of the households had access to improved sources of drinking water in their yards. This finding is almost similar to the report from Sodo Zuria District, Southern Ethiopia, in which 78⋅7 % of the households had access to drinking water from an improved source^(23)^. However, it is by far a higher proportion than that reported in rural Ethiopia, which showed only 29 % of the households having improved source drinking water nearby their dwellings^(42)^. This could be related to the basic difference in the accessibility of safe drinking water between urban and rural settings. About household hygiene, a handwashing facility with soap and water around the toilet was observed only among 10⋅9 % of households. In contrast to this, the 2016 EDHS report documented that a higher proportion of the sample households (52⋅85 %) had handwashing facilities with soap and water around the toilet^(21)^. This big difference in proportion might be related to the respondents’ over-reporting and the larger sample size of the 2016 EDHS national survey. Moreover, the proportion of households (51⋅8 %) that used improved sanitation (safe collection and disposal of the households’ human faeces, and wastes) in the present study was by far lower than that of Nepal (75⋅7 %)^(35)^. The situation is associated with low coverage by the municipality service, careless behaviour and financial constraints of the households for on-time final disposal, as reported by the respondents.

The proportion of caesarean section delivered children (25⋅9 %) was higher than that of the findings (20⋅2 %) reported from Worabe town, Silte Zone^(43)^. We did not find an association between the mode of delivery and children's stunting. This is in contrast to a study from Ghana which discovered that vaginally born children had significantly higher (0⋅121, *P* = 0⋅002) linear growth than their counterparts^(44)^. Another study also indicated that delivery through CS has a positive association with overnutrition rather than undernutrition in the case of elective CS^(45)^. However, in the present study, no such associations were identified, whether in elective or emergency cases. The overall practice of optimum breast-feeding in the present study was 66⋅2 %, which is higher than studies carried out in Bishofitu (8⋅8 %)^(46)^, Hula District, Southern Ethiopia (43⋅1 %)^(47)^ and Worabe (42⋅1 %)^(34)^. This could be because of the educational status of the mothers, as more than 1/3 of them achieved the educational level of secondary school to the College.

In bivariate analysis, antibiotics taken for 1–2 courses within 3 months before the survey was significantly associated with stunting. However, the association was not statistically significant while adjusting for other variables. This might be due to a relatively shorter duration, which could influence the frequency of antibiotics taken, taking the antibiotics as prescribed by the physician, and limitations related to the mother's or caregiver's recall for the frequency of exposure. As pointed out in the LeBrasseur *et al.* study, the effect of antibiotics on the nutritional status of children depends upon the type, number and timing of exposure^(24)^. However, those issues were not considered in our study, which might contribute to the weak association between antibiotic exposure and stunting. Low dietary diversity was positively correlated with stunting in the study area. This is in agreement with many findings in Ethiopia as well as worldwide^(23–37,42)^. Researchers from Burkina Faso reported that having higher dietary diversity at an early age had positively contributed to child height for age^(48)^. Another report from West Guji Zone, Oromia, stated that households that were food insecure were more likely to have stunted children than food-secure ones^(49,50)^. Household food insecurity is one of the factors that contribute to low dietary diversity.

Our findings did not show a significant association between the WaSH components and stunting in children. This is in line with the 2016 EDHS national survey report, which revealed that WaSH components could affect children's stunting as combined not separate. However, a study from the Afar region found that both poor hygiene facilities and no access to improved drinking water sources significantly correlated to stunting^(51)^. The disagreement of the result might be partially explained by the mix-up of rural and urban settings, larger sample size, and not considering sanitation issues that could influence the contribution of drinking water and hygiene. The case of sanitation should get more attention in the pastoralist areas as the households’ living style is highly connected with pet animals, which could also be the cause of some zoonotic diseases. Another WaSH intervention study conducted among the rural households of Ethiopia having under-five children indicated that good hygienic practice among the under-five children and their mothers during the critical moments is more significantly associated with stunting than access to improved drinking water^(23)^. Wondimu pointed out that access to improved drinking water and sanitation are not significantly associated with stunting in Ethiopia when other independent variables are controlled^(52)^. Concerning those findings, our result suggests that strengthening good hygienic practice through the behavioural change of the community accompanied with access to improved drinking water would be more effective in the reduction of stunting in children.

In the present study, maternal education was associated with child stunting, which is a similar finding to other reports^(40,41,43–53)^. This could be explained by the close interaction between mother and child, and the role of formal education in awareness creation regarding hygiene, dietary diversity, child feeding practice and the capability of decision making. According to Alemneh and his colleagues’ findings, households with a mother of formal education had a greater chance of including eggs, an important for early child growth, in the family's diet than illiterate mothers in the rural communities of Ethiopia^(46–54)^. A study on dietary diversity for children under-five in Madagascar underlined that poor maternal education is associated with low dietary diversity for her children^(55)^ which in turn affects the nutritional status of the child^(18,56)^. In the present study, children from female-headed households had a higher chance of being stunted than their counterparts. This is also in harmony with the previous findings in the Amhara region from the data of the 2016 EDHS^(4)^. The predisposing impact of female-headed households on children's stunting might be due to the additional burden on females other than the homemaker role.

## Conclusion

The present study assessed the association between thirteen independent variables and undernutrition among children under five in Hawassa City. The prevalence of overall undernutrition was lower in the study area. Stunting was significantly associated with dietary diversity, maternal educational level and sex of households’ heads. The government sector working on nutrition and public health should focus on enhancing the dietary diversity of households and encouraging women's education. It would certainly be beneficial to emphasise the creation of awareness, at a community level, of the strong links between diet, WaSH and public health.
