# The association of socio-demographic and environmental factors with stunting among under-five children in Hawassa City, Sidama National Regional State, Ethiopia – CORRIGENDUM

**DOI:** 10.1017/jns.2023.85

**Published:** 2023-09-27

**Authors:** B. Kibemo, Afework Mulugeta, Dejene Hailu, Baye Gelaw

Details: correction to tables 1 and 3

Corrected tables supplied

**Table 1.** Socio-demographic characteristics and environment-related factors of child-mother pairs at Hawassa, Sidama Region, Ethiopia (N = 340)

Variables	Category	N (%)
Age in month	6–12	33 (9·7)
	13–35	177 (52·1)
	36–59	130 (38·2)
Sex	Male	171 (50·3)
	Female	169 (49·7)
Mode of delivery	Spontaneous	252 (74·1)
	Cesarean section	88 (25·9)
Breastfeeding practice	Optimum	225 (66·2)
	Suboptimal	115 (33·8)
Antibiotic exposure	Exposed	108 (31·8)
	Not exposed	232 (68·2)
Improved source of drinking water	Yes	301 (88·5)
	No	39 (11·5)
Hand washing facility around the toilet	Improved	37 (10·9)
	Not improved	303 (89·1)
HH's feces and wastes management	Improved	176 (51·8)
	Not improved	164 (48·2)
Marital status	United	318 (93·5)
	Not united	22 (6·5)
	College and above	58 (17·1)
Maternal education level	9–12 grade	72 (21·2)
	8 grade and below	210 (61·8)
	3–4	160 (47·1)
Family size	5–6	139 (40·9)
	7–10	41 (12·1)
	Male	247 (72·6)
Sex of head of household	Female	41 (12·1)
	Headed jointly	52 (15·3)

**Table 3:** Factors associated with stunting among children aged 6–59 months at Hawassa City, Sidama Region, Ethiopia (N = 340)

Variables	Predictors category	COR(95% C.I)	*P*	AOR(95% C.I)	*P*
Age in months	6–12	1		1	
	13–35	0·46 (0·13–1·66)	0·24	0·45 (0·12–1·66)	0·23
	36–59	0·73 (0·39–1·36)	0·32	0·76 (0·4–1·44)	0·39
			
Sex	Male	0·92 (0·5, 1·68)	0·79	0·89 (0·48–1·65)	0·72
	Female	1		1	
Antibiotic exposure	Exposed	1·53 (0·82, 2·84)	0·18	1·65 (0·86–3·18)	0·13
	Not exposed	1		1	
Dietary diversity score (DDS)	<4	3·042 (1·21–7·56)	0·017	2·98 (1·18–7·51)	0·020*
	≥4	1		1	
Maternal education	Illiterate	1		1	
	College& above	0·16 (0·04, 0·69)		0·22 (0·05–0·99)	0·049*
Sex of head of household	Female	1		1	
	Male	0·17 (0·4, 0·65)	0·010	0·16 (0·03, 0·73)	0·018*
Marital status	Not united	1·78 (0·63, 5·08)	0·28	0·59 (0·15, 2·33)	0·46
	United	1		1	
Hand washing facility near the toilet	Not available	3·31 (0·77, 14·21)	0·11	3·1(0·69–13·96)	0·14
	Available	1		1	
Access to drinking water	Not improved	0·76 (0·32, 1·83)	0·54	0·79 (0·32–1·89)	0·63
	Improved	1		1	
Sanitation status	Not improved	1·34 (0·73, 2·46)	0·34	1·38 (0·74–2·55)	0·3
	Improved	1		1	

**Key:** P*= significant at p < 0·05, 1-reference

